# Mitogenomic architecture and phylogenetic placement of *Ctenophthalmus yunnanus* and *Frontopsylla diqingensis*: insights from comparative genomics

**DOI:** 10.3389/fvets.2025.1683581

**Published:** 2025-10-29

**Authors:** Shaobo Tang, Lei Chen, Jun Wu, Bin Chen, Shuang Liu, Mingna Duan, Dandan Jiang, Wei Gu, Quanfu Zhang, Xing Yang

**Affiliations:** ^1^Integrated Laboratory of Pathogenic Biology, College of Preclinical Medicine, Dali University, Dali, China; ^2^The Base for Control and Prevention of Plague and Brucellosis, Chinese Center for Disease Control and Prevention, Baicheng, Jilin, China; ^3^Dali Bai Autonomous Prefecture People's Hospital Ophthalmology, Dali, Yunnan, China; ^4^School of Public Health, Dali University, Dali, China; ^5^Yunnan Provincial Department of Education Key Laboratory of Infectious Diseases, Department of Infection, The First Affiliated Hospital of Dali University, Dali, China; ^6^Department of Gastroenterology, Clinical Medical College and the First Affiliated Hospital of Chengdu Medical College, Key Laboratory of Gastrointestinal Tumors and Microenvironment, Sichuan Provincial Universities, Chengdu, Sichuan, China

**Keywords:** *Ctenophthalmus yunnanus*, *Frontopsylla diqingensis*, fleas, mitochondrial genome, phylogenetic analysis, comparative analyses

## Abstract

Fleas are among the most common hematophagous ectoparasites of mammals. In addition to causing allergic dermatitis and anemia, they can transmit various pathogens. Currently, molecular data on fleas remain relatively scarce. This study sequenced the complete mitochondrial genomes of *Ctenophthalmus yunnanus* (first mitogenome reported) and *Frontopsylla diqingensis* from Yunnan, China, using Illumina sequencing. Comparative analyses with existing flea mitogenomes available in NCBI included nucleotide diversity and selective pressure assessments. Phylogenetic trees were reconstructed based on the PCG123 and PCG12 datasets using the Maximum Likelihood (ML) and Bayesian Inference (BI) methods, respectively. The mitogenomes of *C. yunnanus* (15,801 bp) and *F. diqingensis* (15,878 bp) were circular double-stranded molecules. Both genomes comprised 37 genes. Analysis of the comparative genomic data revealed that most fleas examined possessed mitochondrial genomes approximately 16,000 bp in length, with an average AT content nearing 78%. Additionally, most species exhibited negative AT and GC skews. Among the 13 PCGs, the codons UUA, UUU, and AUU were used most frequently. Analysis of nucleotide diversity and selection pressure indicated that the *cox1* gene exhibited the lowest values for both Pi and Ka/Ks. Phylogenetic analysis demonstrated that the families Ctenophthalmidae and Leptopsyllidae were paraphyletic. Divergence time estimation indicated that the most recent common ancestor of crown-group fleas diverged during the Cretaceous period, while the majority of extant lineages within Siphonaptera underwent diversification following the K-Pg boundary. This study provides valuable mitochondrial genomic data for fleas, which lays a foundation for future genetic and phylogenetic studies and advances our understanding of siphonapteran evolution.

## 1 Introduction

Fleas are obligate hematophagous parasites with a global distribution, whose host range includes wild animals such as rodents and Carnivora, as well as domestic livestock and poultry like dogs, cats, cattle, and sheep. Different flea species exhibit host specificity, while some are capable of cross-host parasitism, acting as critical links for disease transmission (1). As vectors of dual concern in veterinary medicine and public health, they not only directly impair the productivity of livestock and the health of companion animals but also form “animal-to-human” transmission chains through pathogen dissemination. This poses significant risks to regional veterinary prevention and control systems and public health security, causing prominent infestations in farming areas, companion animal communities, and other regions (2). The global annual expenditure on flea control for livestock, poultry, and companion animals exceeds $15 billion (3). In veterinary clinical practice, the harms caused by flea bites are well-documented: susceptible groups such as puppies and lambs may suffer from iron deficiency anemia due to acute blood loss, and even death in severe cases; adult livestock and poultry often develop skin lesions and secondary infections due to pruritus-induced scratching, which reduces milk yield, egg production, and the commercial value of fur-bearing animals; furthermore, salivary allergens from fleas can induce atopic dermatitis in dogs and cats, with its incidence showing an annual upward trend. Fleas are even more pivotal vectors for zoonotic diseases, and the pathogens they carry pose both veterinary prevention priorities and public health risks: *Yersinia pestis* (61) can bridge rodents and humans via domestic dogs and cats, serving as a key vehicle for plague spread; *Bartonella* (62) can circulate in cat populations and is associated with cat-scratch disease cases in humans worldwide; Rickettsia can cause spotted fever in dogs and typhus in humans; in addition, the larvae of *Dipylidium caninum* (Linnaeus, 1758) can utilize fleas to form a “flea-animal-human” parasitic chain, which mainly infects puppies and children (4–7). Flea infestations have constrained the efficiency of livestock farming and compromised the welfare of companion animals. The diseases they transmit often trigger regional epidemics, posing challenges to veterinary epidemic surveillance. The continuous emergence of new and recurrent flea-borne diseases underscores the urgent need to enhance monitoring and research on flea vectors. Consequently, establishing an efficient and precise flea species database will not only provide essential foundational data for the prevention and control of flea-transmitted diseases but will also significantly advance the development of flea biodiversity research and phylogenetic analysis (8).

Currently, the classification and identification of fleas predominantly rely on morphological characteristics (9). However, this approach faces significant challenges in accurately distinguishing closely related and cryptic species. Due to the widespread occurrence of morphological specialization among flea species, coupled with their highly mixed distribution patterns—where multiple flea species can simultaneously parasitize different hosts—significant genetic variation may arise among populations. This phenomenon is particularly pronounced in flea populations that parasitize distinct hosts or are geographically dispersed. Such populations often exhibit subtle morphological differences, which can complicate traditional morphological identification methods and potentially lead to erroneous conclusions regarding species identification (10). The mitochondrial genome serves as an ideal molecular marker because it has significant characteristics, including a compact genome size, highly conserved sequences, and strict maternal inheritance. These features enable it to effectively address the limitations of traditional morphological identification methods, thereby demonstrating unique advantages in flea taxonomy research. In particular, the mitochondrial genome plays a crucial role in overcoming challenges such as differentiating closely related species and reconstructing higher-order phylogenetic relationships. As a result, it has emerged as an indispensable molecular marker, providing essential molecular biological evidence for refining the flea classification system and advancing phylogenetic studies (11–14). However, current research on the mitochondrial genomes of fleas continues to face significant data gaps. Notably, the analysis of the mitochondrial genomes has made considerably less progress. Within the classification system of fleas, representative species from most family and genus-level taxonomic units lack mitochondrial genomic data. This deficiency not only hinders the phylogenetic reconstruction of fleas based on molecular markers but also imposes substantial limitations on accurate species identification. Furthermore, this lack of data complicates in-depth investigations into flea molecular systematics, particularly in critical areas such as higher-order meta-taxonomic relationship analysis and species-level identification, where significant challenges remain.

In this research, (I) we sequenced the mitochondrial genomes of *Ctenophthalmus yunnanus* (60) and *Frontopsylla diqingensis* (15). Subsequent analyses focused on their structural features, base composition, and codon usage preferences. (II) Furthermore, leveraging available mitochondrial genome data of fleas, we assessed nucleotide diversity and evolutionary rates across flea taxa with characterized mitochondrial genomes. (III) Concurrently, we conducted comparative analyses of flea mitochondrial genomes retrieved from the NCBI database, constructed phylogenetic trees using the concatenated nucleotide sequences of all codon positions of 13 protein-coding genes (PCG123) and the concatenated nucleotide sequences of the first and second codon positions of 13 protein-coding genes (PCG12) via maximum likelihood (ML) and Bayesian inference (BI) methods. And ultimately performed divergence time estimation. This not only enriched the flea mitochondrial genome database but also laid a foundation for flea species identification, population genetics, and phylogenetic studies.

## 2 Methods

### 2.1 Sample collection, morphological identification and DNA extraction

A total of 28 specimens of *C. yunnanus* were collected from Gongshan Dulong and Nu Autonomous County in the Nujiang Lisu Autonomous Prefecture of Yunnan Province in China (27°44′21.2″N, 98°40′2.2″E). Additionally, *F. diqingensis* was collected from Fugong County within the same prefecture (26°54′20.8″N, 98°52′4.3″E), comprising 27 individuals. In addition, the samples of these fleas were gathered from the nests of the animals (Statement: This study was approved by the Animal Ethics Committee of Dali University. Application number: 2023-SL-280, Approval number: 2023-P2-280). The collected fleas were immersed in 75% ethanol and then kept in a - 20 °C freezer for subsequent experiments. Flea specimens were identified by professional taxonomists based on morphological characteristics described in “*Fauna Sinica Insecta Siphonaptera*” (15) (*C. yunnanus* possesses a frontal tubercle situated below the midpoint of the frontal margin, with no small notch present between the frontal tubercle and the oral angle. The frontal bristles consist of one row of five bristles. The occipital bristles are arranged in three rows, comprising 2, 2–3, and 5 bristles, respectively. The labial palps are relatively long, extending nearly to the end of the fore coxae. The pronotal comb typically bears 17 spines. The pronotum of the mesothorax has 5–6 pseudosetae on the cervical sclerite. The metepimeron of the metathorax bears five bristles. The fore coxa has 25–30 bristles on the outer surface. The second tarsomere of the hind leg is approximately equal in length to the combined length of the third and fourth tarsomeres. Tergites I-VII each bear two rows of bristles. Tergites II-VII each have one bristle below the spiracle. The apical spinules on tergites I-IV are arranged as follows: 2, 2, 1–2, and 0–1, respectively. *F. diqingensis* possesses a frontal tubercle situated below the midpoint of the frontal margin. The frontal bristles number 6–7. The marginal occipital bristles number 5–7. The labial palps reach or nearly reach the apex of the fore coxa. The pronotal comb consists of 19–22 spines, with the dorsal spines longer than or subequal to the dorsal margin of the pronotum. The metanotum bears 2–3 apical spinules. The metepisternum bears 6–9 bristles arranged in two rows. The outer surface of the hind tibia bears 12–20 bristles arranged in two or three rows. The apical long bristle of the second tarsomere of the hind leg reaches approximately the midpoint of the fourth tarsomere. Tergites I-VII bear 2–3 rows of bristles). Microscopic photography of the diagnostic features of *C. yunnanus* and *F. diqingensis* was performed using an SZ2-ILST dissecting microscope (Olympus, Tokyo, Japan). All type specimens are preserved at the Institute of Pathogen and Vector Biology, Dali University (Collection numbers: DLU230820 and DLU230821). Total genomic DNA was extracted using the TIANamp Genomic DNA Kit (TIANGEN, Beijing, China), adhering to the manufacturer’s protocol.

### 2.2 Sequence, assembly, and annotation analysis

The Illumina NovaSeq 6,000 platform, serviced by Shanghai Biotechnology Co., LTD, was utilized to conduct paired-end (PE) sequencing, with one library generated for each sample, featuring a read length of 150 bp. The raw data acquired through sequencing were counted, and the analysis was conducted employing FastQC software for quality control. To guarantee the quality of the ensuing information analysis, the raw data underwent filtering to produce high-quality sequences (clean data). The A5-miseq v20150522 program was utilized to assemble the complete mitochondrial genome. After sequencing on an Illumina platform, we annotated the genomes with MITOS (16, 17), and then the sequences were blasted against the NCBI database to further confirm the locations of the 37 genes. The secondary structures of 22 tRNA genes in *C. yunnanus* and *F. diqingensis* were predicted using tRNAscan-SE v.2.0 and ARWEN v.1.2.3 (18, 19). The mitochondrial genome sequences of *C. yunnanus* and *F. diqingensis* have been submitted to GenBank under accession numbers OR780664 and OR780662, respectively.

The base composition was calculated using DNAstar v6 software, and the AT and GC skew were then computed using these formulas: AT-skew = (A − T)/ (A + T) and GC-skew = (G − C)/ (G + C). The relative synonymous codon usage (RSCU) of the 13 PCGs was analyzed using CodonW 1.4.2. Furthermore, the DNAsp v6 software was utilized to conduct a sliding-window analysis for assessing nucleotide diversity (20), using a sliding window length of 200 and a step size of 20. This software was further utilized to compute the ratios of non-synonymous (Ka) to synonymous (Ks) substitutions for the 13 PCGs, enabling the analysis of the evolutionary rate through the Ka/Ks ratio.

### 2.3 Phylogenetic analysis

Using *Boreus elegans* (HQ696579) as the outgroup, a phylogenetic analysis was conducted between *C. yunnanus* and *F. diqingensis* investigated in this study and the flea sequences from seven families available in the NCBI database (Supplementary Table S4). Two data matrices were used: (I) the concatenated nucleotide sequences of all codon positions of 13 protein-coding genes (PCG123); and (II) the concatenated nucleotide sequences of the first and second codon positions of 13 protein-coding genes (PCG12). For each matrix, phylogenetic trees were reconstructed using both ML and BI methods. The sequences of the PCG123 and PCG12 datasets were aligned separately using MAFFT. In the ML analysis, the best-fit models were determined using ModelFinder (21): GTR + F + R4 (PCG123 dataset) and GTR + F + I + G4 (PCG12 dataset). ML phylogenetic trees were built in IQ-TREE v.1.6.12, with branch support estimated by 1,000 bootstrap replicates. BI analysis was performed using MrBayes v.3.2.7, running four independent Markov chain Monte Carlo (MCMC) chains for 1,000,000 generations and sampling every 1,000 generations. The first 25% of samples were discarded as burn-in. Finally, the phylogenetic trees were visualized and graphically optimized using the online tool iTOL (https://itol.embl.de/itol.cgi) (22).

### 2.4 Divergence time estimations

Divergence time estimation was performed using BEAST v10.5.0 software, based on the dataset of 13 PCGs from the mitochondrial genomes of flea species in this study. First, ModelFinder was employed to screen for the optimal model for the sequences (21). Subsequently, in the BEAUti module, the optimal substitution model was set as the GTR model, and the Uncorrelated Relaxed Clock model was adopted as the clock model. For the tree prior, the Yule Process was selected, with the Prior Distribution specified as an exponential distribution to facilitate divergence time estimation. Due to the limited fossil record and molecular sequence data of fleas, this study selected the following amber fossil specimens as calibration points to establish the evolutionary timeframe of the order Siphonaptera: (1) The flea genus *Pulex* Linnaeus, 1758: A female flea fossil, *Pulex larimerius*, found in Dominican amber, exhibits highly consistent morphological characteristics with extant members of *Pulex*. Mammalian hairs preserved alongside the fossil provide ecological evidence for host association. Although the fossil is attributed to the Miocene, the geological age of Dominican amber remains highly controversial, with estimated ranges varying between 20 and 15 Mya (Iturralde–Vinent and MacPhee, 1996) and 45–30 Mya (Schlee, 1990). To fully account for this dating uncertainty, the calibration range for this node was set to 35–15 Mya (23, 24). (2) Ctenophthalmidae: Fossil specimens of several species of the genus Palaeopsylla (family Ctenophthalmidae) have been discovered in Baltic amber. Furthermore, the geological age of Baltic amber is consistently determined by the academic community to be 40–35 Mya (25, 26). Thus, the calibration point is set at 40–35 Mya. The MCMC simulation was set to run for 40,000,000 generations, with sampling conducted every 1,000 generations. The Tracer v1.7.2 software was then used to check the ESS values of each parameter, thereby evaluating the reliability of the simulation results. Next, the TreeAnnotator program was executed, where the first 10% of burn-in samples were discarded before generating the divergence time tree. Finally, the constructed divergence time tree was visualized and edited using tvBOT (27).

## 3 Results

### 3.1 The mitochondrial genome characterization of *Ctenophthalmus yunnanus* and *Frontopsylla diqingensis*

**Figure 1:**
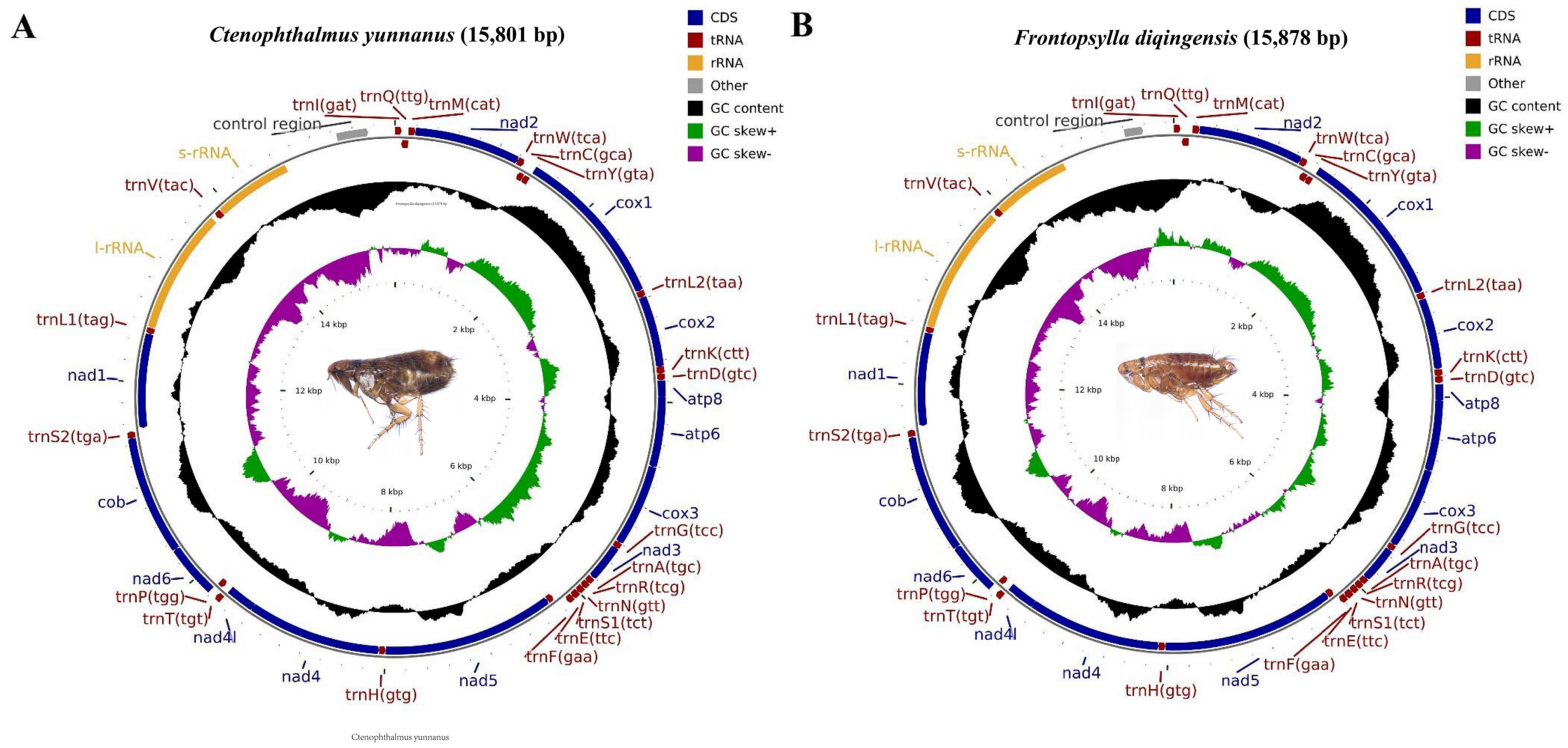
Circular map and organization of the mitochondrial genome of *Ctenophthalmus yunnanus* **(A)** and *Frontopsylla diqingensis* **(B)**.

In this research, we acquired the mitochondrial genomic sequences for *C. yunnanus* and *F. diqingensis*. The characteristics observed are consistent with those of previously reported flea mitochondria, exhibiting a typical closed ring double-stranded structure (28, 29). The lengths of the two genomes are 15,801 bp and 15,878 bp, respectively (Figure 1). Each genome includes a total of 37 genes: 13 PCGs, 22 transfer RNA (tRNA) genes, and 2 ribosomal RNA (rRNA) genes, along with non-coding regions. Out of these 37 genes, nine PCGs (*nad2*, *cox1*, *cox2*, *atp8*, *atp6*, *cox3*, *nad3*, *nad6*, and *cytb*) and 14 tRNAs (*trnI*, *trnM*, *trnW*, *trnL2*, *trnK*, *trnD*, *trnG*, *trnA*, *trnR*, *trnN*, *trnS1*, *trnE*, *trnT*, and *trnS2*) are situated on the H chain. The four remaining PCGs (*nad5*, *nad4*, *nad4L*, and *nad1*), together with eight tRNAs (*trnQ*, *trnC*, *trnY*, *trnF*, *trnH*, *trnP*, *trnL1*, and *trnV*) and two rRNAs (*rrnL* and *rrnS*) are located on the L chain. The mitochondrial genome of *C. yunnanus* contains 13 gene overlapping regions, with a total length of 38 bp (length range: 1–8 bp). The longest overlapping region occurs between *trnW* and *trnC*, measuring 8 bp. Additionally, there are 7 intergenic spacer regions totaling 159 bp (length range: 1–81 bp), with the longest spacer located between *trnE* and *trnF*, measuring 81 bp. In comparison, the mitochondrial genome of *F. diqingensis* features 12 gene overlapping regions, totaling 30 bp (length range: 1–7 bp), with the longest overlapping regions occurring between *atp8* and *atp6*, as well as *nad4* and *nad4L*. There are 13 intergenic spacer regions, totaling 128 bp (length range: 1–31 bp), with the longest spacer situated between *trnQ* and *trnM*, measuring 31 bp (Supplementary Table S1). The nucleotide composition of the mitochondrial genome of *C. yunnanus* is as follows: A = 39.05%, T = 40.31%, G = 7.97%, and C = 12.67%, resulting in A + T = 79.36%, AT-skew = −0.016, G + C = 20.64%, and GC-skew = −0.228. And the mitochondrial genome of *F. diqingensis* has the following nucleotide composition: A = 38.28%, T = 41.05%, G = 8.12%, and C = 12.55%. This results in a combined A + T content of 79.33%, an AT-skew value of −0.035, a G + C content of 20.67%, and a GC-skew of −0.215. Notably, the AT content is considerably greater than the GC content in both flea species, suggesting a strong AT bias (Supplementary Table S2).

### 3.2 Protein-coding genes (PCGs): lengths, start/stop codons, codon usage (RSCU), and base composition/skews

The total lengths of the 13 PCGs in *C. yunnanus* and *F. diqingensis* are 11,118 bp and 11,143 bp, respectively, which account for 70.36 and 70.18% of the total length of their mitochondrial genomes. Among these genes, *nad5* is the longest, measuring 1,714 bp and 1,734 bp for *C. yunnanus* and *F. diqingensis*, respectively, while *atp8* is the shortest, at 159 bp and 162 bp, respectively. Both species use the ATN (N = A, T, G, C) start codon. Whereas TAA is the predominant termination codon, exceptions include: the use of an incomplete T codon in *C. yunnanus* (*cox2*, *nad5*, *nad4*) and *F. diqingensis* (*nad4*); and the use of TAG in *C. yunnanus* (*cytb*) and *F. diqingensis* (*nad3*). In addition, the nucleotide skews of the 13 PCGs of *C. yunnanus* were all negative for AT-skew, with a skew range of −0.187 (*cox3*) to −0.049 (*cox2*), and negative for GC-skew, except for *nad5*, *nad4*, *nad4L*, and *nad1*, with a skew range of - 0.325 (*nad6*) to 0.680 (*nad4L*). Similarly, the nucleotide skews of the 13 PCGs of *F. diqingensis* were all negative for AT-skew as well, with a skew range of −0.252 (*nad3*) to −0.068 (*atp8*), and GC-skew was also negative except for *nad5*, *nad4*, *nad4L* and *nad1*, with a skew range of −0.315 (*nad3*) to 0.591 (*nad4L*) (Supplementary Tables S1, S2).

**Figure 2:**
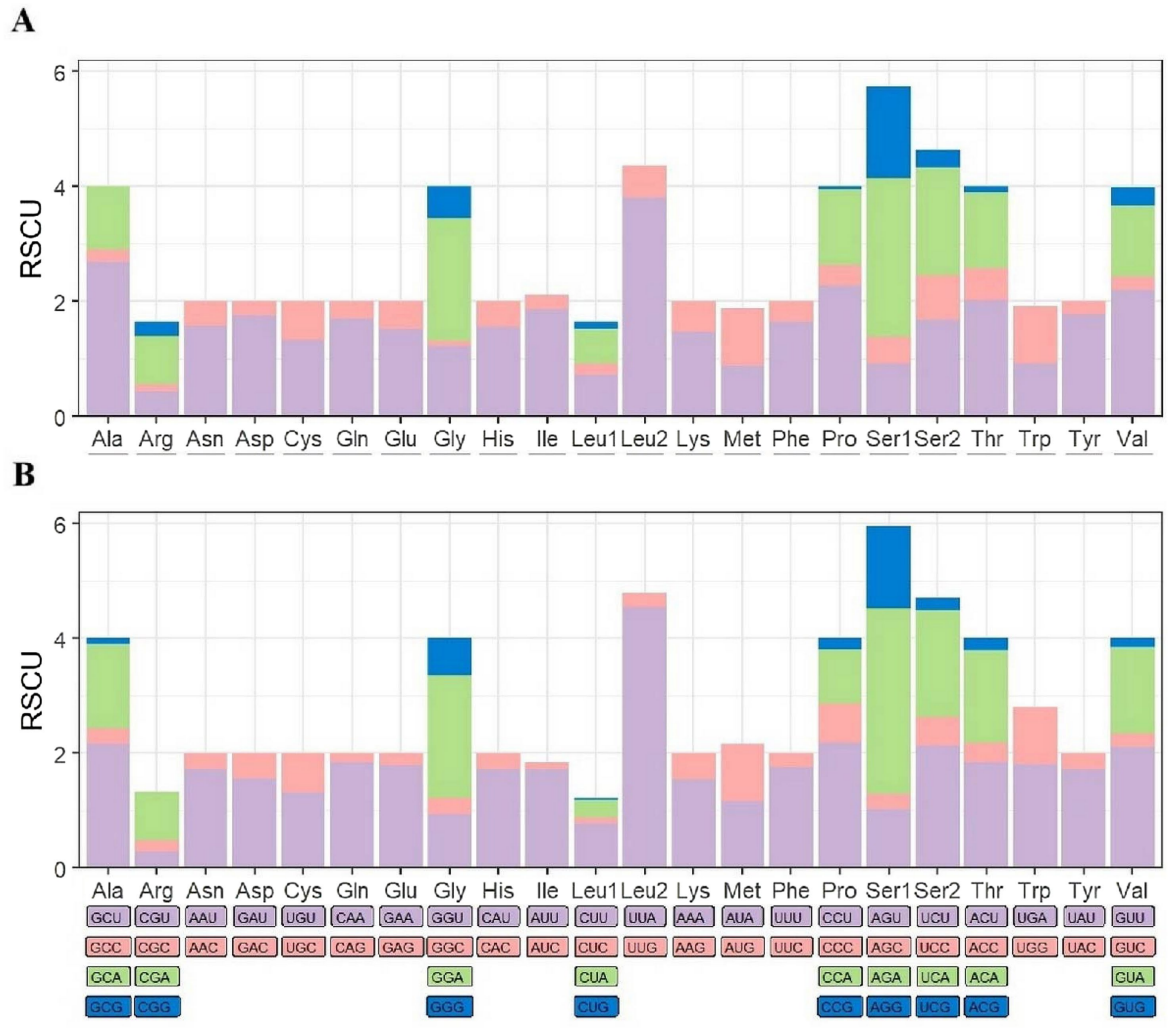
*Ctenophthalmus yunnanus* **(A)** and *Frontopsylla diqingensis* **(B)** protein-coding gene relative synonymous codon usage (RSCU).

In the comparative analysis of 13 PCGs between *C. yunnanus* and *F. diqingensis*, significant differences and similarities in codon usage bias were observed. The mitochondrial genome of *C. yunnanus* encodes a total of 3,544 codons (excluding stop codons), among which 26 codons have an RSCU value greater than 1. The codon UUA exhibits the highest RSCU value of 3.81. The most frequently used codons are AUU, UUU, UUA, AAU, and UAU, with AUU appearing most often (363 times), while GCG is not used at all. In contrast, *F. diqingensis* encodes 3,653 codons (excluding stop codons), also with 26 codons showing an RSCU value above 1. Here, UUA demonstrates an even stronger bias with an RSCU value of 4.54. The most common codons are UUA, AUU, UUU, AUA, and AAU, with UUA being used most frequently (384 times). Similarly, GCG is not utilized. In both species, leucine (Leu), isoleucine (Ile), phenylalanine (Phe), and serine (Ser) are the most abundantly used amino acids. However, differences are noted in the least used amino acids: in *C. yunnanus*, arginine (Arg), glutamine (Gln), and histidine (His) are the least frequent, whereas in *F. diqingensis*, aspartic acid (Asp), cysteine (Cys), glutamine (Gln), and arginine (Arg) are the least utilized (Figure 2; Supplementary Table S3).

### 3.3 Transfer and ribosomal RNA genes (tRNAs and rRNAs): structures and compositional features

Among the 22 tRNAs of *C. yunnanus* and *F. diqingensis*, 14 tRNAs are located on the H chain and 8 tRNAs are located on the L chain. Except for *trnS1*, which lacks the DHU arm, the remaining 21 tRNAs exhibit the typical cloverleaf structure. For *C. yunnanus*, the tRNA length ranges from 61 to 72 bp, with *trnC* being the shortest and *trnK* the longest. The A + T content is 80.07%, and both the AT-skew and GC-skew exhibit positive values. There are a total of 19 base mismatches, with G-U mismatches being the most frequent, occurring 15 times, followed by U–U mismatches, which occur 4 times. As for *F. diqingensis*, the tRNA length ranges from 62 to 70 bp, with *trnG* and *trnL1* as the shortest and *trnK* as the longest. The A + T content for this species is 80.19%, and similarly, both the AT-skew and GC-skew are also positive. The predicted tRNA secondary structures exhibited a total of 21 non-canonical base pairings, comprising 17 G-U wobble pairs and 4 U–U mismatches (Supplementary Tables S1, S2 and Supplementary Figure S1).

The rRNA genes of *C. yunnanus* and *F. diqingensis* comprise both *rrnL* and *rrnS*, with the *trnV* gene located between them. The length of the *16S rRNA* in *C. yunnanus* is 1,251 bp, whereas the *12S rRNA* is 780 bp long. The AT content for the *16S rRNA* and the *12S rRNA* stands at 81.85 and 80.90%, respectively, and with both the negative AT-skew and positive GC-skew. In *F. diqingensis*, the *16S rRNA* has a length of 1,281 bp, with the *12S rRNA* measuring 786 bp. The AT content for the *16S rRNA* in this species is 82.98%, while for the *12S rRNA*, it is 81.30%. Additionally, both the AT-skew and GC-skew values for these two rRNAs are positive (Supplementary Tables S1, S2).

### 3.4 Comparative mitochondrial genomics analysis of the fleas

**Figure 3:**
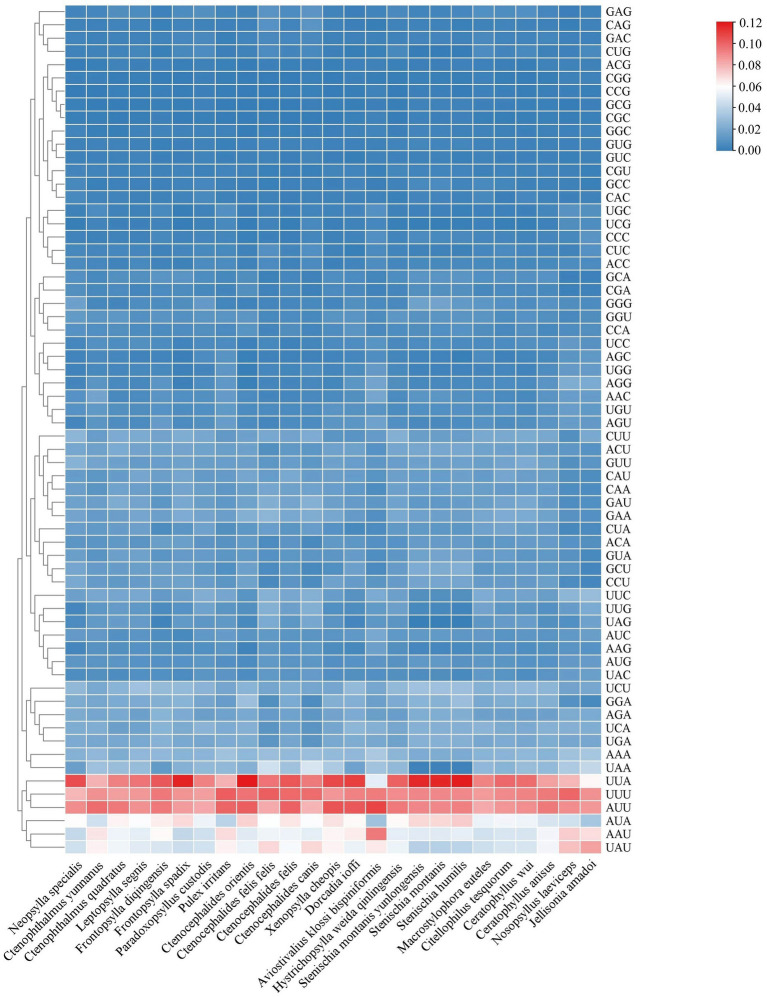
Heat map of codon usage frequency.

In this study, we compared *C. yunnanus* and *F. diqingensis* with the mitochondrial genomes of the flea species published on NCBI in terms of genome length, AT and GC content, base skew of the nucleotide sequence and the codon usage frequency of the PCGs. We found that the mitochondrial genome lengths of most flea species are approximately 16,000 bp. However, we observed that *Pulex irritans*, *Ctenocephalides orientis*, *C. felis felis*, *C. felis*, *Xenopsylla cheopis*, and *Ceratophyllus wui* possess significantly longer mitochondrial genomes compared to other flea species. In terms of AT content and base skew of nucleotide sequences, the majority of flea species examined in this study exhibited an AT content of approximately 78%. However, the AT content for *C. orientis* (83.21%), *C. felis felis* (82.88%), *C. felis* (83.13%), and *X. cheopis* (82.83%) was significantly higher than that of the other flea species. Furthermore, we observed that, except for the AT-skew (0.001) of *Neopsylla specialis* and both the AT-skew (0.024) and GC-skew (0.248) of *Leptopsylla segnis* were positive, the AT-skew and GC-skew were negative for the remaining flea species included in this study (Supplementary Table S4). Additionally, we analyzed the codon usage frequency of the PCGs across these flea species. The analysis revealed that among the 13 PCGs, the codons UUA, UUU, and AUU were utilized more frequently (Figure 3).

### 3.5 Nucleotide diversity analysis and non-synonymous/synonymous (Ka/Ks) substitution ratio

**Figure 4:**
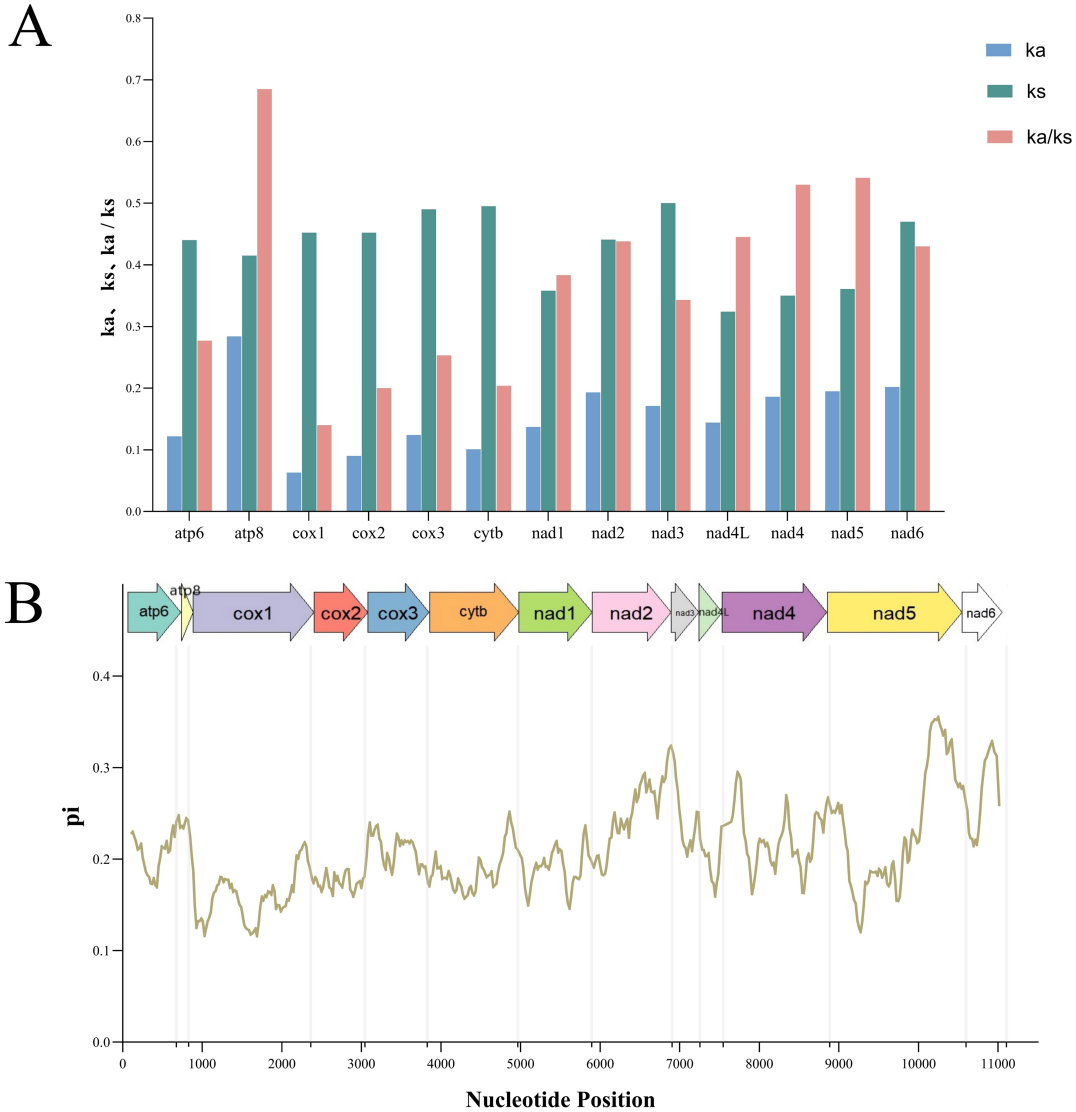
Non-synonymous (Ka) to synonymous (Ks) ratios of 13 protein-coding genes in mitochondrial genomes of the order Siphonaptera **(A)**. And nucleotide diversity (Pi) of mitochondrial genomes of the flea species from the order Siphonaptera. The size of the sliding window is 200 bp with each step of 20 bp. The arrows above the figure indicate protein-coding genes **(B)**.

Analysis of the 13 PCGs revealed that the Ka/Ks ratio was less than 1 for all genes. Among them, *atp8* exhibited the highest Ka/Ks value, followed by *nad5*, *nad4*, *nad4L*, and *nad2*, whereas *cox1* had the lowest. This pattern indicates that all 13 PCGs are under purifying selection (Figure 4A). Additionally, *nad5*, *nad6*, and *nad2* displayed the highest nucleotide diversity (Pi > 0.30), while *cox1* exhibited the lowest Pi value (Pi < 0.13) (Figure 4B).

### 3.6 Phylogenetic analysis

**Figure 5:**
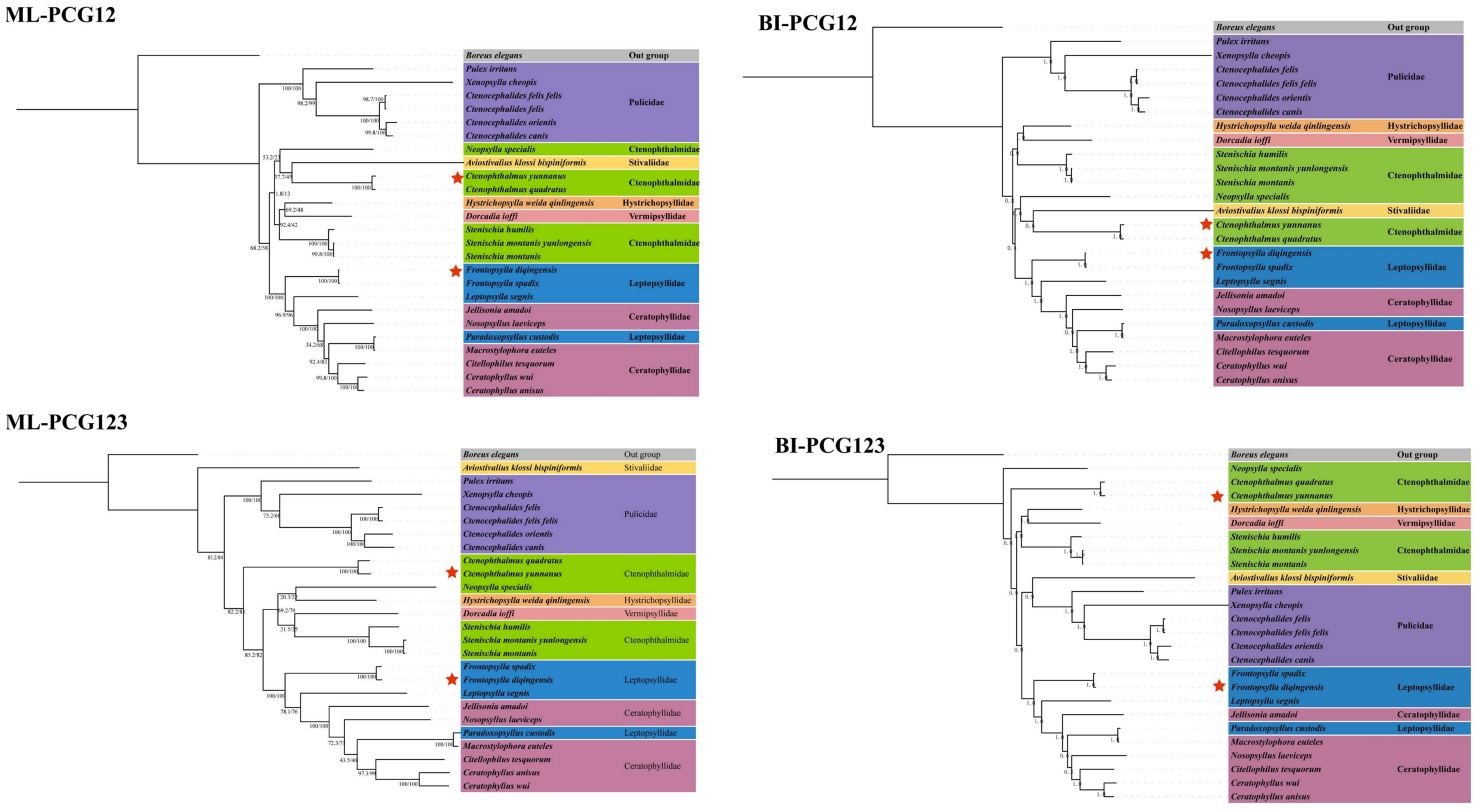
Phylogenetic reconstruction of Siphonaptera based on PCG123 and PCG12 datasets using Bayesian inference and Maximum likelihood. *Boreus elegans* (HQ696579) was used as the outgroup. The numbers beside the nodes are posterior probabilities (BI) and SH-Alrt/UFboot (ML) indicates the species in this study.

Using *Boreus elegans* (HQ696579) as the outgroup, four phylogenetic trees were constructed based on the PCG123 and PCG12 datasets, respectively, using the ML method and BI method (Figure 5). The analysis results showed that the family Pulicidae stably formed a monophyletic group in all phylogenetic trees, while the families Ctenophthalmidae, Leptopsyllidae, and Ceratophyllidae all exhibited obvious paraphyletic characteristics. In addition, the flea species of Leptopsyllidae and Ceratophyllidae were concentrated in the same clade, suggesting a close phylogenetic relationship between the two families. In all four trees, *F. diqingensis* and *F. spadix* always clustered together with high support values, indicating their close phylogenetic relationship; *C. yunnanus* and *C. quadratus* also clustered into one clade with high support values, which showed that each of these two species had a close phylogenetic relationship.

### 3.7 Divergence time estimation

**Figure 6:**
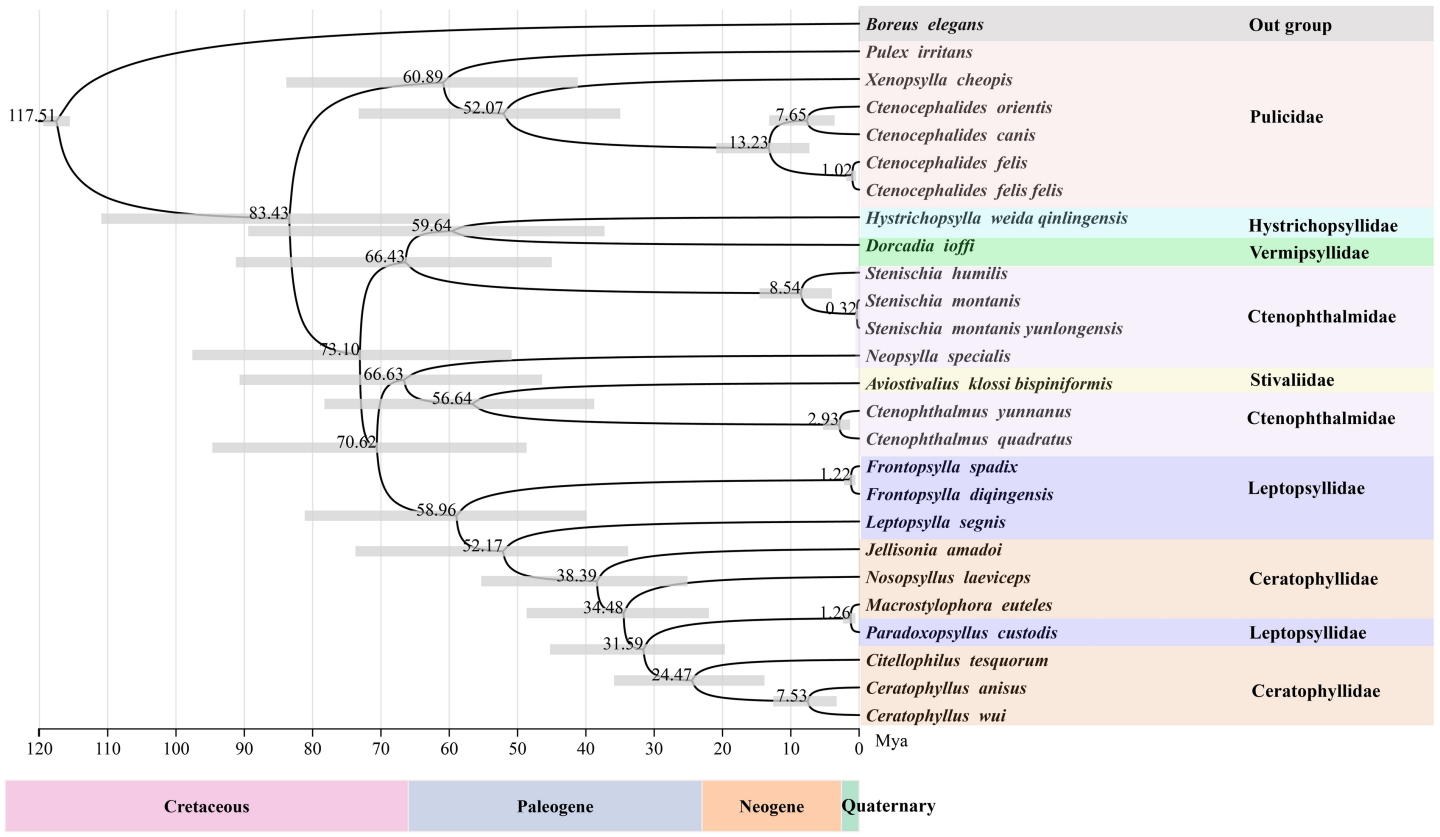
Divergence time estimation for the various lineages within the order Siphonaptera (node labels represent the mean values of the estimated ages, with node bars indicating 95% confidence intervals).

According to divergence time estimation results, the most recent common ancestor of extant Siphonaptera dates back to the Cretaceous period, although the major intraordinal divergence events occurred after the K-Pg boundary. This study reveals that the family Pulicidae diverged from a clade comprising Ctenophthalmidae, Leptopsyllidae, Vermipsyllidae, Stivaliidae, Hystrichopsyllidae, and Ceratophyllidae approximately 83.43 Mya. Among them, Ctenophthalmidae, to which *C. yunnanus* belongs, began to diverge around 66.43 Mya, while Leptopsyllidae, which includes *F. diqingensis*, initially diverged approximately 58.96 Mya (Figure 6).

## 4 Discussion

Fleas are a widespread group of obligate ectoparasites that cause multifaceted harm to their hosts through biting and blood-feeding activities. On one hand, they can directly trigger clinical conditions such as allergic dermatitis, pruritus, alopecia, and anemia, significantly compromising host health and welfare. On the other hand, and more importantly, fleas serve as vectors for a variety of zoonotic pathogens-including Rickettsia, Bartonella, plague bacterium, and tapeworms-thereby substantially increasing the risk of cross-species transmission of infectious diseases between animals and humans. This poses persistent and complex challenges to regional and even global public health security. Furthermore, flea infestations lead to reduced productivity in livestock and poultry, increased veterinary costs for companion animals, and rising expenses for control measures, collectively resulting in extensive and substantial economic losses (2, 30, 31). Therefore, accurate identification of flea species is essential. Nevertheless, depending exclusively on morphological traits, the host, and geographical distribution when identifying fleas can present difficulties. Such methods are limited by environmental changes and sample size. In contrast, mitochondrial DNA, characterized by its small genome, high conservatism, maternal inheritance, and rapid evolutionary rate, serves as an effective molecular marker to enhance the accuracy of flea species identification (32).

In this research, we conducted the analysis of the mitochondrial genomes of *C. yunnanus* and *F. diqingensis*. A comparative analysis was performed on the mitochondrial genomes of flea species that have been published and are accessible on NCBI, focusing on the mitochondrial genome features, nucleotide diversity, selective pressure, and phylogenetic relationships. The results of this study show that the stop codons of most PCGs are typical ones (UAA and UAG); meanwhile, some genes are also detected to use incomplete stop codons (T). Notably, these incomplete stop codons are converted into functional stop codons through post-transcriptional polyadenylation (19, 33–35). Research has revealed the presence of base mismatches in tRNA genes, with G-U mismatches being the most prevalent. Previous studies have indicated that such mismatches are predominantly enriched at the junction regions between the single-stranded V loop and the TΨC helix of tRNA. This structural characteristic may arise from the sharp turn of the polynucleotide chain at this location, where G-U pairing effectively stabilizes the transition of this backbone conformation. Notably, G-U mismatches in tRNA genes not only contribute to the formation of their higher-order structures but also exhibit a high degree of conservation throughout evolution. Despite the presence of mismatches, these sites can be corrected through RNA editing mechanisms, thereby not affecting the normal transport function of tRNA. These features hold significant biological importance for maintaining the structural stability and functional specificity of tRNA molecules (36, 37).

The RSCU values for *C. yunnanus* and *F. diqingensis* were calculated. And the number of codons with RSCU value >1 in both fleas was 26, and both of them had the largest RSCU value of UUA. An RSCU value of less than 1 demonstrates a weak preference for codons, whereas an RSCU of exactly 1 reflects no preference, conversely, an RSCU greater than 1 shows a strong preference for codon usage (38). This suggests that UUA is the strongest preference for use among the 13 PCGs of the two flea species studied. Additionally, it was noted that the majority of codons terminating with A or U exhibited RSCU values exceeding 1, whereas those concluding with G or C showed RSCU values under 1, which is consistent with patterns found in other metazoans (39). The analysis of nucleotide diversity revealed that the *cox1* gene exhibits the lowest Pi value. In the analysis of selective pressures, the Ka/Ks ratio is used to assess whether there is selective pressure on PCGs. Specifically, a Ka/Ks ratio greater than 1 suggests positive selection, which implies that the gene might be undergoing adaptive changes. A ratio of 1 is considered neutral selection, indicating that the gene’s evolution is not affected by natural selection. In contrast, a Ka/Ks ratio less than 1 indicates purifying selection, meaning that the gene is likely to maintain its original function and minimize harmful mutations (40). Therefore, among the 13 PCGs in the mitochondrial genome examined in this study, the Ka/Ks ratios were all less than 1, indicating that these 13 PCGs are under purifying selection. Additionally, in both nucleotide diversity analysis and selection pressure analysis, the *cox1* gene exhibited the lowest pi value and Ka/Ks ratio, suggesting that it demonstrates the lowest level of variation and evolutionary rate. Consequently, this gene fragment can also be applied to taxa with closely related, ambiguous, and highly variable morphological characteristics (13, 41, 42). Moreover, it has long been recognized as a universal barcode for species identification (43, 44).

In relation to the study of the phylogenetic connections within the order of fleas, the research team led by Whiting et al. (45) performed phylogenetic analyses utilizing four distinct gene fragments: 18S rDNA, 28S rDNA, COII, and EF1-*α*. These analyses incorporated 128 representative flea species globally, encompassing 16 families and 83 genera. To deduce evolutionary relationships, both the direct optimization method and the ML method were utilized. Their results demonstrate that at the section-level classification unit, Tungidae, Lycopsyllidae, Pygiopsyllidae, Stivaliidae, Stephanociricidae, Rhopalopsyllidae, Pulicidae, Ceratophyllidae, Ischnopsyllidae, and Chimaeropsyllidae form monophyletic groups. Conversely, Hystrichopsyllidae, Ctenophthalmidae, and Leptopsyllidae show distinct paraphyletic traits (45). However, Whiting et al.’s study was based solely on four single gene fragments (18S rDNA, 28S rDNA, COII, and EF1-α), which inherently limited the phylogenetic signals obtained. Recognizing this constraint, the authors themselves emphasized the necessity of incorporating new molecular data to clarify the systematic classification and phylogeny of fleas. This study confirmed the monophyly of Pulicidae, along with the paraphyletic nature of Ctenophthalmidae and Leptopsyllidae. The results obtained are largely in accordance with those from many earlier studies (14, 28, 46, 47). Notably, the results of this study indicate that the family Ceratophyllidae exhibits a paraphyletic characteristic. This finding is consistent with the majority of previous mitochondrial genome-based studies, whereas it contradicts the conclusions of Whiting et al. However, due to the current scarcity of flea mitochondrial genome data, this gap significantly limits the depth of phylogenetic studies on fleas. Therefore, it is essential to continuously expand the availability of mitochondrial genomic data for fleas. Meanwhile, since nuclear genes can provide more reliable and comprehensive evolutionary signals when resolving the evolutionary relationships of higher - rank taxa, the integration of nuclear genome data will also be indispensable for clarifying deep - level phylogenetic relationships in future studies.

The phylogenetic relationships between Siphonaptera and other insect orders have long been a focal point of debate and research in entomological systematics. As an independent branch on the insect phylogenetic tree, the order Siphonaptera is supported by various morphological characteristics, including lateral flattening, winglessness and piercing-sucking mouthparts, as well as its unique living habits. In the 19th century, some scholars posited that fleas were closely related to beetles based on their external morphology (48). However, in the late 20th century, Willi Hennig (49) demonstrated that fleas are closely related to scorpionflies (Mecoptera) and true flies (Diptera), together constituting the group Antliophora. Phylogenetic analyses using *18S* and *28S rRNA*, along with cox2 and EF-1α genes, revealed that the order Mecoptera is polyphyletic, with the order Siphonaptera being a branch of Mecoptera. Additionally, Siphonaptera is recognized as a sister group to the family Boreidae, while the family Nannochoristidae is placed as a sister group to the clade comprising Boreidae and Siphonaptera (50–53). In 2020, Tihelka et al. (54) selected 26 representative species from the group Antliophora, encompassing all suborders of the order Mecoptera, Siphonaptera and Diptera. Additionally, based on transcriptome data previously collected by the “Evolution of a Thousand Insect Transcriptomes (1KITE)” team, 1,478 directly homologous single-copy protein-coding nuclear genes were retrieved. The researchers also constructed smaller data matrices that included the mitochondrial genomes of 29 species in conjunction with multiple genes. The research results indicate that the order Siphonaptera is nested within the order Mecoptera and exhibits a sister group relationship with the family Nannochoristidae (54). Furthermore, a phylogenetic analysis conducted in 2022 by Yu Zhang et al. based on mitochondrial genomes reveals a sister relationship between Siphonaptera and the clade comprising Diptera, Mecoptera, Megaloptera, and Neuroptera (29).

Both the present study and the work by Yu Zhang et al. employed mitochondrial genomes for phylogenetic reconstruction, yet with distinct research foci: our study specifically addresses the phylogenetic relationships within Siphonaptera species, while Zhang et al.’s investigation centers on the phylogenetic position of Siphonaptera relative to Antliophora insects. Notably, both studies share a common limitation: incomplete taxonomic sampling across flea lineages. Although compelling evidence from various studies has demonstrated both the genetic distinctiveness and specific phylogenetic patterns among different flea species within Siphonaptera, as well as between Siphonaptera and Antliophora, these findings require further validation through additional research. Mitochondrial genomes serve as highly effective molecular markers, offering dual utility for both precise flea species identification and comprehensive phylogenetic analyses. Nevertheless, current data availability remains critically limited, as evidenced by the NCBI database containing fewer than 30 complete mitochondrial genomes representing the entire order Siphonaptera (55–59). Therefore, future studies incorporating a broader representation of Siphonaptera mitochondrial genomes, with extensive sampling across diverse species, will be crucial for elucidating the phylogenetic relationships among different flea species within Siphonaptera, as well as the phylogenetic position of this order relative to Antliophora. Meanwhile, the findings of this study also provide important guidance for flea control practices. Significant differentiation exists among flea lineages in terms of ecological adaptability, host preference, and vector competence, and such functional divergence in an evolutionary context directly influences their disease transmission potential. Therefore, elucidating the phylogenetic status and evolutionary trajectory of key vector flea species will not only help reveal the evolutionary mechanisms underlying their transmission-related traits but also offer a theoretical basis for targeted control strategies.

This study estimates that the most recent common ancestor of the extant flea crown-group could be traced back to the Cretaceous. The establishment of this key time node provides an important calibration point for reconstructing the evolutionary history of fleas. It is noteworthy that although the ancestral lineages of fleas had already formed in the Cretaceous, molecular clock analyses reveal that the substantial divergence events of most extant lineages occurred predominantly after the K-Pg boundary. This finding is consistent with the research conclusions of Zhu et al., which not only confirms that flea evolution is significantly correlated with geological historical events but also reveals the crucial role of the K-Pg extinction event in the formation of flea diversity (24). From an evolutionary biology perspective, this pattern of divergence timing strongly suggests that the adaptive radiation of mammals (particularly rodents and chiropterans) after the K-Pg boundary likely created new niche opportunities for fleas, thereby promoting their subsequent lineage divergence. This discovery provides new empirical evidence for understanding the co-evolutionary patterns between hosts and parasites on a macro-evolutionary scale. However, given that this study utilized mitochondrial genome data and the current scarcity of definitive flea fossils, potential errors in the divergence time estimation cannot be ruled out; mitochondrial genomes evolve faster than nuclear genomes, a characteristic that makes them more suitable for studies at lower taxonomic ranks but limits their application in research involving higher taxonomic ranks. Therefore, expanding species representation and geographic coverage is expected to facilitate a clearer resolution of the evolutionary history and diversification patterns of this group.

## 5 Conclusion

This research presents the assembly of the mitochondrial genomes of *C. yunnanus* and *F. diqingensis*. In both nucleotide diversity analysis and selection pressure analysis, the *cox1* gene exhibited the lowest values for Pi and Ka/Ks. Additionally, phylogenetic analysis indicated that both the family Ctenophthalmidae, which includes *C. yunnanus*, and the family Leptopsyllidae, which includes *F. diqingensis*, are paraphyletic. Divergence time estimation indicates that the most recent common ancestor of crown-group fleas diverged during the Cretaceous period, while the majority of extant lineages within Siphonaptera underwent diversification following the K-Pg boundary. This research not only enriches the mitochondrial genome database of fleas but also provides valuable insights for flea genetics and phylogenetic studies. However, the absence of mitochondrial genome data for all flea lineages necessitates the acquisition of additional mitochondrial genome data to address taxonomic issues and controversies across various orders of fleas, thereby aiding in the formulation of more effective prevention and control strategies.

## Data Availability

The nucleotide sequences of the *C. yunnanus* and *F. diqingensis* mitogenome were deposited in NCBI (https://www.ncbi.nlm.nih.gov/) under accession number OR780664 and OR780662, respectively.
