# Supplementary material for: Mitogenomic architecture and phylogenetic placement of Ctenophthalmus yunnanus and Frontopsylla diqingensis: insights from comparative genomics
Source: Front Vet Sci. 2025 Oct 29;12:1683581. doi: 10.3389/fvets.2025.1683581 (PMC12605306; doi:10.3389/fvets.2025.1683581)
Supplement: Supplementary file 1 [file Table_1.doc]

***Supplementary Material***

**1 Supplementary Tables and Figures**

**1.1 Supplementary Tables**

**Table S1** Mitochondrial genome structure characteristics of *Ctenophthalmus yunnanus* / *Frontopsylla diqingensis*.

| **Gene** | **Strand** | **Positions** | **Size (bp)** | **Initiation codon** | **Termination codon** | **Anticodon** | **Intergenic nucleotides** |
| --- | --- | --- | --- | --- | --- | --- | --- |
| *trnI* | H | 1-62 / 1-63 | 62 / 63 |  |  | GAT |  |
| *trnQ* | L | 128-60 / 146-78 | 69 / 69 |  |  | TTG | -3 / 14 |
| *trnM* | H | 128-193 / 178-245 | 66 / 68 |  |  | CAT | -1 / 31 |
| *nad2* | H | 194-1,204 / 246-1,256 | 1,011 / 1,011 | ATT / ATT | TAA / TAA |  | 0 / 0 |
| *trnW* | H | 1,203-1,267 / 1,255-1,319 | 65 / 65 |  |  | TCA | -2 / -2 |
| *trnC* | L | 1,320-1,260 / 1,385-1,319 | 61 / 67 |  |  | GCA | -8 / -1 |
| *trnY* | L | 1,385-1,321 / 1,448-1,386 | 65 / 63 |  |  | GTA | 0 / 0 |
| *cox1* | H | 1,383-2,921 / 1,446-2,981 | 1,539 / 1,536 | ATC / ATC | TAA / TAA |  | -3 / -3 |
| *trnL2* | H | 2,922-2,984 / 2,986-3,049 | 63 / 64 |  |  | TAA | 0 / 4 |
| *cox2* | H | 2,985-3,660 / 3,051-3,731 | 676 / 681 | ATT / ATG | T / TAA |  | 0 / 1 |
| *trnK* | H | 3,661-3,732 / 3,734-3,803 | 72 / 70 |  |  | CTT | 0 / 2 |
| *trnD* | H | 3,732-3,793 / 3,803-3,868 | 62/66 |  |  | GTC | -1 / -1 |
| *atp8* | H | 3,794-3,952 / 3,878-4,039 | 159 / 162 | ATC / ATA | TAA / TAA |  | 0 / 9 |
| *atp6* | H | 3,946-4,617 / 4,033-4,707 | 672 / 675 | ATG / ATG | TAA / TAA |  | -7 / -7 |
| *cox3* | H | 4,617-5,399 / 4,707-5,489 | 783 / 783 | ATG / ATG | TAA / TAA |  | -1 / -1 |
| *trnG* | H | 5,400-5,462 / 5,490-5,551 | 63 / 62 |  |  | TCC | 0 / 0 |
| *nad3* | H | 5,463-5,813 / 5,552-5,902 | 351 / 351 | ATT / ATT | TAA / TAG |  | 0 / 0 |
| *trnA* | H | 5,818-5,880 / 5,901-5,965 | 63 / 65 |  |  | TGC | 4 / -2 |
| *trnR* | H | 5,880-5,941 / 5,964-6,027 | 62 / 64 |  |  | TCG | -1 / -2 |
| *trnN* | H | 5,942-6,005 / 6,028-6,092 | 64 / 65 |  |  | GTT | 0 / 0 |
| *trnS1* | H | 6,006-6,074 / 6,093-6,161 | 69 / 69 |  |  | TCT | 0 / 0 |
| *trnE* | H | 6,075-6,138 / 6,162-6,226 | 64 / 65 |  |  | TTC | 0 / 0 |
| *trnF* | L | 6,281-6,220 / 6,289-6,225 | 62 / 65 |  |  | GAA | 81 / -2 |
| *nad5* | L | 7,995-6,282 / 8,023-6,290 | 1,714 / 1,734 | ATA / ATG | T / TAA |  | 0 / 0 |
| *trnH* | L | 8,058-7,996 / 8,090-8,025 | 63 / 66 |  |  | GTG | 0 / 1 |
| *nad4* | L | 9,394-8,059 / 9,426-8,091 | 1,336 / 1,336 | ATG / ATG | T / T |  | 0 / 0 |
| *nad4L* | L | 9,681-9,388 / 9,713-9,420 | 294 / 294 | ATG / ATG | TAA / TAA |  | -7 / -7 |
| *trnT* | H | 9,684-9,747 / 9,716-9,780 | 64 / 65 |  |  | TGT | 2 / 2 |
| *trnP* | L | 9,810-9,748 / 9,843-9,781 | 63 / 63 |  |  | TGG | 0 / 0 |
| *nad6* | H | 9,822-10,331 / 9,855-10,361 | 510 / 507 | ATT / ATA | TAA / TAA |  | 11 / 11 |
| *cytb* | H | 10,331-11,467 / 10,361-11,500 | 1,137 / 1,140 | ATG / ATG | TAG / TAA |  | -1 / -1 |
| *trnS2* | H | 11,466-11,528 / 11,504-11,569 | 63 / 66 |  |  | TGA | -2 / 3 |
| *nad1* | L | 12,482-11,547 / 12,522-11,590 | 936 / 933 | ATG / ATG | TAA / TAA |  | 18 / 20 |
| *trnL1* | L | 12,545-12,484 / 12,585-12,524 | 62 / 62 |  |  | TAG | 1 / 1 |
| *rrnL* | L | 13,796-12,546 / 13,866-12,586 | 1,251 / 1,281 |  |  |  | 0 / 0 |
| *trnV* | L | 13,906-13,839 / 13,962-13,896 | 68 / 67 |  |  | TAC | 42 / 29 |
| *rrnS* | L | 14,685-13,906 / 14,747-13,962 | 780 / 786 |  |  |  | -1 / -1 |
| control region |  | 15,248-15,545 / 15,407-15,584 | 298 / 178 |  |  |  |  |

**Table S2** Nucleotide composition and skewness of mitochondrial genomes of *Ctenophthalmus yunnanus* / *Frontopsylla diqingensis*.

| **Region** | **A%** | **T%** | **G%** | **C%** | **A + T%** | **G + C%** | **AT-skew** | **GC-skew** |
| --- | --- | --- | --- | --- | --- | --- | --- | --- |
| **Whole genome** | 39.05 / 38.28 | 40.31 / 41.05 | 7.97 / 8.12 | 12.67 / 12.55 | 79.36 / 79.33 | 20.64 / 20.67 | -0.016 / -0.035 | -0.228 / -0.215 |
| ***nad2*** | 35.71 / 35.21 | 47.28 / 46.88 | 6.33 / 7.32 | 10.68 / 10.58 | 82.99 / 82.10 | 17.01 / 17.90 | -0.139 / -0.142 | -0.256 / -0.182 |
| ***cox1*** | 30.02 / 29.23 | 40.61 / 40.89 | 13.97 / 14.26 | 15.4 / 15.63 | 70.63 / 70.12 | 29.37 / 29.88 | -0.150 / -0.166 | -0.049 / -0.046 |
| ***cox2*** | 35.95 / 35.24 | 39.64 / 41.56 | 10.06 / 9.99 | 14.35 / 13.22 | 75.59 / 76.80 | 24.41 / 23.20 | -0.049 / -0.082 | -0.176 / -0.139 |
| ***atp8*** | 38.99 / 42.59 | 46.54 / 48.77 | 5.03 / 3.09 | 9.43 / 5.56 | 85.53 / 91.36 | 14.47 / 8.64 | -0.088 / -0.068 | -0.304 / -0.286 |
| ***atp6*** | 34.38 / 32.89 | 42.71 / 44.89 | 9.23 / 9.33 | 13.69 / 12.89 | 77.08 / 77.78 | 22.92 / 22.22 | -0.108 / -0.154 | -0.195 / -0.160 |
| ***cox3*** | 29.63 / 30.91 | 43.3 / 41.89 | 12.64 / 12.39 | 14.43 / 14.81 | 72.92 / 72.80 | 27.08 / 27.20 | -0.187 / -0.151 | -0.066 / -0.089 |
| ***nad3*** | 32.76 / 29.63 | 46.73 / 49.57 | 9.4 / 7.12 | 11.11 / 13.68 | 79.49 / 79.20 | 20.51 / 20.80 | -0.176 / -0.252 | -0.083 / -0.315 |
| ***nad5*** | 35.76 / 36.62 | 44.4 / 43.02 | 12.95 / 12.57 | 6.88 / 7.79 | 80.16 / 79.64 | 19.84 / 20.36 | -0.108 / -0.080 | 0.306 / 0.235 |
| ***nad4*** | 33.68 / 34.81 | 45.96 / 44.16 | 12.87 / 13.55 | 7.49 / 7.49 | 79.64 / 78.97 | 20.36 / 21.03 | -0.154 / -0.118 | 0.265 / 0.288 |
| ***nad4L*** | 34.01 / 39.12 | 48.98 / 45.92 | 14.29 / 11.90 | 2.72 / 3.06 | 82.99 / 85.03 | 17.01 / 14.97 | -0.180 / -0.080 | 0.680 / 0.591 |
| ***nad6*** | 39.22 / 34.71 | 45.1 / 49.31 | 5.29 / 5.72 | 10.39 / 10.26 | 84.31 / 84.02 | 15.69 / 15.98 | -0.070 / -0.174 | -0.325 / -0.284 |
| ***cytb*** | 31.13 / 31.93 | 42.92 / 41.40 | 10.56 / 10.70 | 15.39 / 15.96 | 74.05 / 73.33 | 25.95 / 26.67 | -0.159 / -0.129 | -0.186 / -0.197 |
| ***nad1*** | 32.91 / 32.05 | 45.94 / 45.98 | 14.42 / 14.90 | 6.73 / 7.07 | 78.85 / 78.03 | 21.15 / 21.97 | -0.165 / -0.179 | 0.364 / 0.356 |
| ***rrnL*** | 40.37 / 43.09 | 41.49 / 39.89 | 12.31 / 11.71 | 5.84 / 5.31 | 81.85 / 82.98 | 18.15 / 17.02 | -0.014 / 0.039 | 0.357 / 0.376 |
| ***rrnS*** | 39.36 / 40.84 | 41.54 / 40.46 | 12.82 / 11.96 | 6.28 / 6.74 | 80.9 / 81.30 | 19.1 / 18.70 | -0.027 / 0.005 | 0.342 / 0.279 |
| **tRNAs** | 40.78 / 40.44 | 39.29 / 39.75 | 11.17 / 11.05 | 8.76 / 8.76 | 80.07 / 80.19 | 19.93 / 19.81 | 0.019 / 0.009 | 0.121 / 0.116 |

**Table S3** Codon usage in the mitochondrial genome of *Ctenophthalmus yunnanus* and *Frontopsylla diqingensi*

| Codon | Count | Codon | Count | Codon | Count | Codon | Count |
| --- | --- | --- | --- | --- | --- | --- | --- |
| UUU(F) | 328/352 | UCU(S) | 74 / 106 | UAU(Y) | 235 / 180 | UGU(C) | 46 / 35 |
| UUC(F) | 72/49 | UCC(S) | 33 / 25 | UAC(Y) | 30 / 29 | UGC(C) | 23 / 19 |
| UUA(L) | 296/384 | UCA(S) | 82 / 92 | UAA(*) | 115 / 50 | UGA(W) | 71 / 91 |
| UUG(L) | 43 / 22 | UCG(S) | 13 / 11 | UAG(*) | 47 / 11 | UGG(W) | 19 / 21 |
| CUU(L) | 55 / 65 | CCU(P) | 50 / 58 | CAU(H) | 43 / 58 | CGU(R) | 11 / 8 |
| CUC(L) | 16 / 9 | CCC(P) | 8 / 18 | CAC(H) | 12 / 10 | CGC(R) | 3 / 6 |
| CUA(L) | 47 / 25 | CCA(P) | 29 / 25 | CAA(Q) | 40 / 46 | CGA(R) | 21 / 24 |
| CUG(L) | 9 / 3 | CCG(P) | 1 / 5 | CAG(Q) | 7 / 4 | CGG(R) | 6 / 0 |
| AUU(I) | 363 / 353 | ACU(T) | 62 / 64 | AAU(N) | 247 / 228 | AGU(S) | 40 / 51 |
| AUC(I) | 48 / 27 | ACC(T) | 17 / 12 | AAC(N) | 67 / 38 | AGC(S) | 20 / 13 |
| AUA(M) | 170 / 238 | ACA(T) | 41 / 56 | AAA(K) | 99 / 101 | AGA(S) | 69 / 92 |
| AUG(M) | 39 / 33 | ACG(T) | 3 / 7 | AAG(K) | 36 / 30 | AGG(S) | 40 / 41 |
| GUU(V) | 66 / 55 | GCU(A) | 51 / 58 | GAU(D) | 58 / 42 | GGU(G) | 40 / 37 |
| GUC(V) | 7 / 6 | GCC(A) | 4 / 7 | GAC(D) | 8 / 12 | GGC(G) | 3 / 11 |
| GUA(V) | 37 / 39 | GCA(A) | 21 / 39 | GAA(E) | 51 / 59 | GGA(G) | 70 / 85 |
| GUG(V) | 10 / 4 | GCG(A) | 0 / 3 | GAG(E) | 16 / 7 | GGG(G) | 18 / 25 |

**Table S4** Comparative mitochondrial genomics analysis of the fleas

| Family | Species | Size (bp) | A | T | A + T (%) | AT skew | G | C | G+C(%) | GC skew | GenBank accession number |
| --- | --- | --- | --- | --- | --- | --- | --- | --- | --- | --- | --- |
| Ctenophthalmidae | *Neopsylla specialis* | 16820 bp | 38.64 | 38.63 | 77.27 | 0.001 | 8.51 | 14.22 | 22.73 | -0.251 | NC_073019 |
|  | *Ctenophthalmus yunnanus* | 15801 bp | 39.05 | 40.31 | 79.36 | -0.016 | 7.97 | 12.67 | 20.64 | -0.228 | OR780664 |
|  | *Ctenophthalmus quadratus* | 15938 bp | 39.19 | 40.26 | 79.45 | -0.013 | 7.96 | 12.59 | 20.55 | -0.225 | NC_072692 |
|  | *Stenischia montanis yunlongensis* | 15651 bp | 38.20 | 39.10 | 77.29 | -0.012 | 8.66 | 14.05 | 22.71 | -0.237 | OR780663 |
|  | *Stenischia montanis* | 15889 bp | 38.28 | 39.25 | 77.54 | -0.013 | 8.57 | 13.90 | 22.46 | -0.237 | PP990561 |
|  | *Stenischia humilis* | 15617 bp | 38.57 | 39.43 | 78.00 | -0.011 | 8.38 | 13.62 | 22.00 | -0.238 | NC_073020 |
| Leptopsyllidae | *Leptopsylla segnis* | 15785 bp | 40.37 | 38.51 | 78.89 | 0.024 | 13.17 | 7.94 | 21.11 | 0.248 | NC_072691 |
|  | *Frontopsylla diqingensis* | 15878 bp | 38.28 | 41.05 | 79.33 | -0.035 | 8.12 | 12.55 | 20.67 | -0.214 | OR780662 |
|  | *Frontopsylla spadix* | 15085 bp | 37.99 | 40.84 | 78.83 | -0.036 | 8.31 | 12.85 | 21.17 | -0.215 | NC_073018 |
|  | *Paradoxopsyllus custodis* | 15375 bp | 38.10 | 38.69 | 76.79 | -0.008 | 8.60 | 14.61 | 23.21 | -0.259 | OQ627398 |
| Pulicidae | *Pulex irritans* | 20337 bp | 38.92 | 41.09 | 80.02 | -0.027 | 8.53 | 11.45 | 19.98 | -0.146 | NC_063709 |
|  | *Ctenocephalides orientis* | 22189 bp | 39.48 | 43.73 | 83.21 | -0.051 | 6.23 | 10.56 | 16.79 | -0.258 | NC_073009 |
|  | *Ctenocephalides felis felis* | 20911 bp | 39.61 | 43.27 | 82.88 | -0.044 | 6.53 | 10.59 | 17.12 | -0.237 | MW420044 |
|  | *Ctenocephalides felis* | 20873 bp | 39.72 | 43.41 | 83.13 | -0.044 | 6.50 | 10.37 | 16.87 | -0.229 | MT594468 |
|  | *Ctenocephalides canis* | 15609 bp | 38.59 | 39.93 | 78.52 | -0.017 | 8.79 | 12.69 | 21.48 | -0.182 | NC_063710 |
|  | *Xenopsylla cheopis* | 18902 bp | 40.98 | 41.85 | 82.83 | -0.011 | 6.69 | 10.49 | 17.17 | -0.221 | MW310242 |
| Vermipsyllidae | *Dorcadia ioffi* | 16785 bp | 40.10 | 40.61 | 80.71 | -0.006 | 7.74 | 11.55 | 19.29 | -0.198 | NC_036066 |
| Stivaliidae | *Aviostivalius klossi bispiniformis* | 16593 bp | 39.49 | 39.55 | 79.04 | -0.001 | 8.70 | 12.26 | 20.96 | -0.170 | OR774970 |
| Hystrichopsyllidae | *Hystrichopsylla weida qinlingensis* | 17173 bp | 39.10 | 41.49 | 80.59 | -0.030 | 7.56 | 11.85 | 19.41 | -0.221 | NC_042380 |
| Ceratophyllidae | *Macrostylophora euteles* | 16027 bp | 38.49 | 39.10 | 77.59 | -0.008 | 8.20 | 14.21 | 22.41 | -0.268 | NC_085274 |
|  | *Citellophilus tesquorum* | 15345 bp | 37.90 | 40.18 | 78.07 | -0.029 | 8.58 | 13.35 | 21.93 | -0.218 | NC_088096 |
|  | *Ceratophyllus wui* | 18081 bp | 37.72 | 38.99 | 76.71 | -0.017 | 9.51 | 13.78 | 23.29 | -0.183 | NC_040301 |
|  | *Ceratophyllus anisus* | 15875 bp | 38.41 | 40.14 | 78.54 | -0.022 | 8.25 | 13.21 | 21.46 | -0.231 | NC_073017 |
|  | *Nosopsyllus laeviceps* | 16533 bp | 37.93 | 40.17 | 78.10 | -0.029 | 9.14 | 12.76 | 21.90 | -0.165 | PP838812 |
|  | *Jellisonia amadoi* | 17031 bp | 38.78 | 40.39 | 79.17 | -0.020 | 7.61 | 12.95 | 20.83 | -0.260 | NC_022710 |

**1.2 Supplementary Figures**





**Figure S1**  Secondary structure of 22 tRNA genes from the *Ctenophthalmus yunnanus* (A) and *Frontopsylla diqingensis* (B).
